# Influence of the moving fluoroscope on gait patterns

**DOI:** 10.1371/journal.pone.0200608

**Published:** 2018-07-13

**Authors:** Marina Hitz, Pascal Schütz, Michael Angst, William R. Taylor, Renate List

**Affiliations:** Institute for Biomechanics, ETH Zurich, Switzerland; University of Illinois at Urbana-Champaign, UNITED STATES

## Abstract

Video-fluoroscopic analysis can provide important insights for the evaluation of outcome and functionality after total knee arthroplasty, allowing the in vivo assessment of tibiofemoral kinematics without soft tissue artefacts. To enable measurement of the knee throughout activities of daily living such as gait, robotic systems like the moving fluoroscope have been developed that follow the knee movement and maintain the joint in front of the image intensifier. Since it is unclear whether walking while being accompanied by moving fluoroscope affects normal gait, the objective of this study was to investigate its influence on gait characteristics in healthy subjects. In addition, the impact of the motors’ noise was analysed.

By means of skin markers analysis (VICON MX system, Oxford Metrics Group, UK) and simultaneous measurement of ground reaction forces (Kistler force plates, Kistler, Switzerland), gait characteristics when walking with and without the moving fluoroscope as well as with and without ear protectors in combination with the moving fluoroscope, were obtained in young (n = 10, 24.5y ± 3.0y) and elderly (n = 9, 61.6y ± 5.3y) subjects during level gait and stair descent. Walking with the moving fluoroscope significantly decreased gait velocity in level gait and stair descent over the respective movement without the fluoroscope. Statistical analysis, including gait velocity as a covariate, resulted in no differences on the ground reaction force parameters. However, some kinematic parameters (ankle, knee and hip ranges of motion, minimal knee angle in late stance phase, maximal knee angles in stance and swing phase) seemed to be modified by the presence of the moving fluoroscope, but statistical comparison was limited due to velocity differences between the conditions. Wearing ear protectors to avoid the influence of motor sound during walking with the moving fluoroscope caused no significant difference.

Walking with the moving fluoroscope has been shown to decrease gait velocity and small alterations in kinematic parameters were observed. Therefore, gait and movement alterations due to the moving fluoroscope cannot completely be excluded. However, based on the absence of differences in ground reaction force parameters (when adjusted for velocity within ANCOVA), as well as based on the comparable shape of the angular curves to the slow control condition, it can be concluded that changes in gait when walking with the moving fluoroscope are small, especially in comparison to natural slow walking. In order to allow assessment of joint replacement with the moving fluoroscope, including an understanding of the effects of joint pain, clinical analyses can only be compared to gait activities showing similarly reduced velocities. Importantly, the reduced gait speeds observed in this study are similar to those observed after total knee arthroplasty, suggesting that analyses in such subjects are appropriate. However, the moving fluoroscope would likely need to be optimized in order to detect natural gait characteristics at the higher gait velocities of healthy young subjects.

The moving fluoroscope can be applied for comparisons between groups measured with the moving fluoroscope, but care should be taken when comparing data to subjects walking at self-selected speed without the moving fluoroscope.

## Introduction

Video-fluoroscopy in combination with 2D/3D registration allows an accurate quantification of 3D joint motion free of soft tissue artefact and has thus become a well-accepted imaging technique for the acquisition of kinematic information of single joints during functional movement tasks. Research using single plane video-fluoroscopic analysis [1–7], as well as dual orthogonal fluoroscopy [8–10], has provided valuable information on the three-dimensional motion of total knee arthroplasties (TKAs) and healthy knees. However, due to the limited field of view of the stationary image intensifier, static systems can only be applied for the analysis of highly restricted movements [11, 12]. To overcome these limitations, mobile devices such as the robotic radiographic imaging platform [13, 14], the mobile fluoroscopy system [15], the mobile biplane X-ray imaging system [9], and the moving fluoroscope [3, 6, 7, 16], have all been developed to allow the tracking of the knee during complete gait cycles of level gait, stair and ramp walking.

The moving fluoroscope consists of a fluoroscopic unit mounted on a moving trolley that moves with the subject in real-time, and which is controlled by a wire sensor attached to the knee. When the subject’s knee moves, the moving fluoroscope then follows the joint in real time, in both the horizontal and vertical directions, such that the knee remains in the field of view of the image intensifier. Although the ability of the moving fluoroscope to keep the knee in the field of view of the image intensifier has been demonstrated [7], it still remains unknown whether the physical presence of the moving fluoroscope and the sound of the moving fluoroscope’s motors and the visual information from the laboratory may have an influence on the time-distance, kinetic and kinematic parameters of a subject when being tested. Here, as the auditory and motor systems, as well as premotor regions, are known to interact, and especially sounds can have the ability to influence our motor behaviour [17], the noise of the fluoroscope could influence the subject’s gait. As a result, although each subject is provided sufficient time and practice trials to become accustomed to the moving fluoroscope prior to testing, distractions caused by proximity to the large dynamic device could still result in unnatural or disturbed gait patterns.

Yamokoski and Banks (14) have tested the influence of their close-up robot tracking system on gait and found that the dynamic robot tracking caused significant changes in several parameters, such as stride length, ankle sagittal plane rotation as well as anterio-posterior and mediolateral ground reaction forces, although the differences were small and not clinically relevant.

Previous research has shown that rhythmic sensory cues affect temporal dynamics in human gait, but the influence of auditory signals on gait parameters is known to be larger than visual rhythmic cues [18]. However, the reported effects of metronomic cueing on gait velocity and stride length are not consistent [18, 19]. Furthermore, the influence of auditory stimuli seems to be age dependent: In elderly subjects the structure of gait variability can be manipulated using auditory stimulation, as opposed to young people, who seem to dedicate less attentional resources to auditory stimuli [20]. Although rhythmic sensory cues such as music or a metronome affect some parameters of gait, it remains unclear whether the motor sounds of the moving fluoroscope have an impact on natural human gait.

To assess if the moving fluoroscope influences natural gait patterns, the objective of this research project was to analyse the impact of the moving fluoroscope on the gait characteristics, specifically the time distance parameters, whole body kinematics, and ground reaction forces of young and elderly subjects. Additionally, the impact of an acoustic masking intervention with ear protectors was tested. It was hypothesized that none of the kinematic and the ground reaction force parameters differ between conditions.

## Materials and methods

### Subjects

Overall 19 subjects participated in the study, comprising of ten subjects between 20 and 35 years old (24.5y ± 3.0y, five male and five female, BMI 22.0 ± 2.3) and nine subjects older than 55 years (61.6y ± 5.3y, six male and three female, BMI 25.9 ± 4.0) (no drop-outs). An elderly and a young age group were included due to age dependency of the influence of auditory stimuli [20]. Recruitment took place between January and June 2016 using public placards, verbal announcements in different sports and music clubs, as well as in lectures of the study program Health Sciences and Technology. Inclusion criteria were the two defined age ranges and the ability to perform the motion tasks. Subjects with actual significant problems, such as current pain, current injuries of the lower extremities or implants in the lower extremities were excluded from this study, as well as subjects who had already experienced walking with the moving fluoroscope. The study was approved by the ethics committee of ETH Zurich, Switzerland (EK-2015-N-68) and all subjects provided written informed consent prior to participation in the study.

### Data acquisition

The subjects performed two different motion tasks, level gait and stair descent, with and without the moving fluoroscope. Within the moving fluoroscope condition, trials including an acoustic masking intervention were performed. The order of the tasks and intervention were randomized for all subjects, such that for each subject the first task (level gait or stair descent) was randomly selected and for each task it was randomly determined whether the ear protectors were worn or not after the first control condition (Table 1).

**Table 1:** Data acquisition course of action.

Condition	Task	Moving fluoroscope	Acoustic intervention		
Familiarization trials	level gait	x			
Control 1	level gait				randomized
FluMo	level gait	x		randomized	
FluMo intervention	level gait	x	x		
Control 2	level gait				
Slow Control	level gait				
Control 1	stair descent				
FluMo	stair descent	x		randomized	
FluMo intervention	stair descent	x	x		
Control 2	stair descent				

To familiarize the subjects with the device, three level gait practice trials with the moving fluoroscope were performed. Before and after performing all tasks including the moving fluoroscope, control trials without the moving fluoroscope were recorded at a self-selected gait speed (control 1, control 2). Between the control measurements, the condition with the moving fluoroscope (FluMo) and the intervention condition with the moving fluoroscope and an acoustic intervention (FluMo intervention) were performed. For each of the conditions and tasks, five valid gait cycles were conducted. A gait cycle was considered valid when each force plate was hit once only. In addition, the subjects were asked to perform two slow level gait trials (slow control condition) after the second level gait control condition. The slow walking trials were also based on a self-selected gait speed, with subjects requested to walk relatively slower (resulting in an average velocity reduction of 30.3±11.4% to the Control 1 and 24.6±12.6% to the Control 2 conditions, with velocity changes for all subjects ranging from a reduction of 56.4% to an increase of 5.2% in comparison to the two control conditions, n.b. only a single subject exhibited a velocity increase).

Whole body kinematics were assessed using an opto-electronic motion capture system (VICON MX system, Oxford Metrics Group, UK) consisting of 22 infrared cameras and 55 reflective skin markers according to the IfB marker set [21]. Five force plates (Kistler, Instrumentation, Winterthur, Switzerland) embedded but decoupled from the surrounding floor of the movement analysis laboratory were used to measure the ground reaction forces [6, 7]. Additionally, a three step staircase was instrumented with two mobile force plates (Kistler, Instrumentation, Winterthur, Switzerland) [7]. Kinematic and ground reaction force data were recorded simultaneously at 100 Hz and 2000 Hz respectively.

The moving fluoroscope consists of a fluoroscopic unit (BV Pulsera, Philips Medical Systems Switzerland) mounted on a stand-alone robot that follows the knee in the horizontal and vertical directions in real-time, and which is controlled by a wire sensor attached to the knee such that the knee always remains in the field of view of the image intensifier [7]. Since the aim of this study was purely to assess possible kinematic changes in the subject’s interaction with the fluoroscope, no X-ray radiation was involved in this study.

For the acoustic intervention, an ear protector (3M PELTOR WS Alert XP) with a built-in Bluetooth function was used. In order to communicate with the participants, the ear protector was connected via Bluetooth to a microphone. To completely drown any sound of the moving fluoroscope’s motors, white noise was constantly played in the ear protectors.

### Data processing

#### Time distance parameters

Gait events were defined based on the ground reaction forces with a threshold of 25N. The step length (based on the distance the heel marker moved between two consecutive heel strikes) and the cadence of both legs (ipsilateral: leg of the tracked knee; contralateral: leg of non-tracked knee) were determined. The stride velocity was calculated as the stride length divided by the time between two consecutive heel strikes.

#### Kinematics

Four basic motion tasks were used to functionally determine the joint axes and centres [21]. Joint rotations were determined based on redundant marker clusters and a helical axis approach [22]. For clinically interpretable rotational components, the attitude vector was decomposed along the axes of the marker based joint coordinate system fixed in the proximal segments [21]. Only the sagittal plane rotations were compared between the different conditions. All kinematic data were normalized over a gait cycle [23].

The ranges of motion (ROMs) for the ankle *a_ROM*, knee *k_ROM* and hip *h_ROM* joints of both legs were defined as the maximal ranges occurring throughout the whole gait cycle. In addition, the first peak flexion in the stance phase *k_max1* and the second peak flexion in the swing phase *k_max3* of the ipsilateral knee and the minimal ipsilateral knee flexion in the late stance phase *k_min2* were investigated.

#### Ground reaction forces

For the ground reaction force analysis, the first *F*_*z2*_ and second peaks *F*_*z4*_, the minimal ground reaction force between the peaks *F*_*z3*_, the loading rate *b*_*n*_ and unloading rate *e*_*n*_ of the vertical ground reaction forces (based on the definition of Stüssi and Debrunner [24] using the 80% value of *F*_*z2*_ and *F*_*z4*_ to calculate the slope of the force after touchdown, respectively before take-off) were evaluated for level gait [25], whereas in stair descent only the first peak *F*_*z2*_ of the vertical ground reaction force was compared. Furthermore, the maximal posterior *F*_*ymax*_ and maximal anterior ground reaction forces *F*_*ymin*_ were examined.

#### Left-right asymmetry

To check for asymmetries between two consecutive steps of the ipsi- and the contralateral legs during level gait, the absolute symmetry index (ASI) [25] was analysed for the ground reaction force parameters *F*_*z2*_, *F*_*z3*_, *F*_*z4*_, as well as for the time distance parameters stride velocity, step length, and cadence. A critical level of 10% was chosen to differentiate between a symmetric and an asymmetric behaviour [26, 27].

$$ASI\;(\%)=\left|\frac{X_{ipsi}-X_{contra}}{(X_{ipsi}+X_{contra})\times 0.5}\right|\times 100$$ (1)

where *X* represents the parameter in question.

All calculations were performed using MATLAB (Version R2014a, MathWorks, Natick, MA, USA).

### Statistical analysis

A linear mixed factors ANOVA was used to evaluate whether the gait velocity differed between the conditions in both tasks. Two separate Pearson’s bivariate correlation analyses, one each for kinematic and ground reaction force parameters, were undertaken to not only reduce the number of dependent variables in order to reduce the family-wise error rate, but also to avoid investigating essentially the same (or highly correlated) aspects of walking. To additionally identify the kinematic and ground reaction force parameters that are velocity dependent, a further Pearson’s bivariate correlation analysis was conducted. A linear mixed factors ANCOVA was then performed with walking velocity as a covariate for comparisons with the velocity dependent parameters *k_max1*, *k_max3*, *h_ROM*, *k_ROM*, *a_ROM*, *F*_*z2*_, *F*_*z3*_, *F*_*z4*_, *b*_*n*_, *e*_*n*_, *F*_*ymin*_ *and F*_*ymax*_ in level gait, and *h_ROM*, *k_ROM*, *F*_*z2*_, *b*_*n*_, *F*_*ymin*_ *and F*_*ymax*_ in stair descent as the dependent variable. These parameters, which were within the ANCOVA adjusted for gait velocity (ANCOVA with velocity as a covariate), were compared between the conditions at the 25^th^, 50^th^ and 75^th^ percentiles of walking velocity, as well as the mean velocity. For the velocity independent parameters *k_min2* in level gait and *a_ROM*, *k_max1*, *k_min2 and k_max3* in stair descent, linear mixed factors ANOVAs were executed. In both the ANCOVA and the ANOVA models, the independent variables were age (with two levels young and old), condition (with 5 levels for each condition in level gait and 4 levels each condition in stair descent) and interaction age and condition. All p-values for fixed effects were adjusted for multiple comparisons using Holm-Bonferroni ranking correction. The *post hoc* comparisons were conducted using Least Significant Differences (LSDs). Since in level gait, all ground reaction force parameters correlated, only the parameter *F*_*z2*_ was tested for condition and age dependencies, whereas in stair descent, besides *F*_*z2*_ also *F*_*ymin*_ was included in the statistical condition and age analysis, because these parameters did not correlate. Similarly, since cadence and step length correlated with stride velocity, the condition and age analysis was only performed for the time distance parameter stride velocity. Since no significant main effects of age, nor a significant interaction effect of age and condition were found for any parameters, except *k_max1*, the effect of condition was based on all 19 subjects (except for *k_max1*). For *k_max1*, the post hoc comparisons were performed for both age groups separately.

All statistical analyses were performed using IBM SPSS software version 23 (SPSS AG, Zurich, Switzerland) and the significance level was set at *p*<0.05.

Furthermore, to depict the repeatability of waveforms within a test day for each execution form, the coefficient of multiple correlation (CMC) [28] was calculated over all trials of each subject for all kinematic as well as ground reaction force patterns.

## Results

### Time distance parameters

For level gait, the mean stride velocities in the FluMo conditions were significantly lower (28%-32%) than in the control conditions, but did not significantly differ compared to the slow control condition (Table 2). Similarly, during stair descent, gait velocity was significantly decreased in the FluMo conditions compared to the control conditions (19%-22% in young, 11%-13% in old age group) (Table 2).

**Table 2:** Time distance and kinematic parameters.

task	condition	group	stride velocity				step length			Cadence			a_ROM				k_ROM				h_ROM				k_max1			k_min2				k_max3			
			[m/s]				[m]			[1/min]			[°]				[°]				[°]				[°]			[°]				[°]			
**level gait**	Control 1	y	1.27	±	0.10		0.70	±	0.06	109.58	±	2.52	32.4	±	5.8		69.8	±	1.9		44.4	±	3.5		19.0	±	5.9	3.0	±	3.6		69.7	±	4.5	
		o	1.25	±	0.15		0.70	±	0.04	104.40	±	9.26	32.3	±	2.2		68.3	±	5.9		47.4	±	4.6		22.1	±	5.5	2.9	±	2.2		66.0	±	6.0	
	Control 2	y	1.18	±	0.11		0.67	±	0.05	106.06	±	7.24	32.8	±	5.9		67.7	±	2.8		42.4	±	2.2		17.4	±	5.9	3.7	±	3.8		68.3	±	5.2	
		o	1.10	±	0.08		0.67	±	0.05	99.39	±	7.59	30.6	±	2.7		67.0	±	5.6		45.0	±	4.1		20.2	±	4.7	3.4	±	2.2		64.8	±	5.8	
	FluMo	y	**0.92**	**±**	**0.11**	^**a**^^**,**^^**b**^	0.58	±	0.05	95.59	±	6.99	**26.6**	**±**	**3.8**	^**a**^^**,**^^**b**^^**,**^^**c**^	**62.3**	**±**	**4.9**	^**a**^^**,**^^**b**^^**,**^^**c**^	**37.6**	**±**	**1.9**	^**a**^^**,**^^**b**^^**,**^^**c**^	13.2	±	6.4	**5.3**	**±**	**4.3**	^**a**^^**,**^^**b**^^**,**^^**c**^	**63.6**	**±**	**7.0**	^**a**^^**,**^^**b**^^**,**^^**c**^
		o	**0.89**	**±**	**0.08**		0.58	±	0.03	91.64	±	9.71	**27.1**	**±**	**1.7**		**61.5**	**±**	**7.3**		**40.7**	**±**	**2.8**		**18.6**	**±**	**5.3** ^**c**^	**5.6**	**±**	**3.2**		**61.3**	**±**	**8.7**	
	FluMo int.	y	**0.91**	**±**	**0.11**	^**a**^^**,**^^**b**^	0.58	±	0.05	94.47	±	7.03	**26.3**	**±**	**3.5**	^**a**^^**,**^^**b**^^**,**^^**c**^	**62.2**	**±**	**5.3**	^**a**^^**,**^^**b**^^**,**^^**c**^	**38.2**	**±**	**2.1**	^**a**^^**,**^^**b**^	13.1	±	6.8	**4.9**	**±**	**4.1**	^**a**^^**,**^^**b**^^**,**^^**c**^	**63.9**	**±**	**7.1**	^**a**^^**,**^^**b**^
		o	**0.85**	**±**	**0.09**		0.57	±	0.05	89.41	±	8.61	**27.2**	**±**	**2.5**		**62.6**	**±**	**5.7**		**40.1**	**±**	**2.8**		**17.9**	**±**	**4.9** ^**c**^	**5.4**	**±**	**3.7**		**62.2**	**±**	**6.9**	
	Slow control	y	**0.91**	**±**	**0.10**	^**a**^^**,**^^**b**^	0.59	±	0.04	92.72	±	7.69	31.0	±	5.2		**65.7**	**±**	**2.9**	^**a**^	**38.6**	**±**	**1.9**	^**a**^	14.3	±	5.5	3.8	±	3.8		66.3	±	4.9	
		o	**0.84**	**±**	**0.12**		0.60	±	0.06	84.80	±	10.25	27.9	±	2.3		**64.1**	**±**	**5.5**		**41.6**	**±**	**4.1**		14.6	±	4.8	3.3	±	3.3		61.9	±	6.2	
**stair descent**	Control 1	y	0.56	±	0.05		0.32	±	0.02	103.65	±	9.40	55.8	±	4.5		93.3	±	3.8		23.4	±	3.8		34.4	±	5.3	29.1	±	6.2		97.7	±	3.9	
		o	0.51	±	0.03		0.32	±	0.01	95.42	±	6.53	57.3	±	4.7		95.9	±	4.7		24.0	±	3.6		32.8	±	5.4	26.8	±	6.4		97.3	±	6.5	
	Control 2	y	0.54	±	0.03		0.32	±	0.02	102.88	±	7.73	55.8	±	4.0		94.1	±	3.8		23.6	±	3.7		34.6	±	5.5	27.8	±	5.8		98.6	±	4.3	
		o	0.51	±	0.04		0.32	±	0.02	95.65	±	8.53	58.5	±	3.4		96.6	±	4.8		24.2	±	3.4		32.7	±	4.5	26.5	±	4.4		97.5	±	7.0	
	FluMo	y	**0.47**	**±**	**0.04**	^**a**^^**,**^^**b**^	0.32	±	0.02	87.73	±	5.86	56.9	±	3.9		89.6	±	4.2		21.8	±	3.8		32.1	±	5.6	26.2	±	5.4		**95.6**	**±**	**4.1**	^**a**^^**,**^^**b**^
		o	**0.45**	**±**	**0.04**		0.32	±	0.02	84.27	±	7.11	58.0	±	3.6		93.8	±	3.0		23.0	±	2.8		32.6	±	4.5	25.9	±	6.0		**96.7**	**±**	**6.3**	
	FluMo int.	y	**0.46**	**±**	**0.04**	^**a**^^**,**^^**b**^	0.32	±	0.02	86.50	±	7.93	57.3	±	3.6		89.8	±	4.8		22.8	±	3.9		31.6	±	5.5	25.5	±	5.5		**95.6**	**±**	**4.6**	^**a**^^**,**^^**b**^
		o	**0.46**	**±**	**0.04**		0.32	±	0.01	86.53	±	7.09	58.3	±	3.1		93.4	±	3.2		22.6	±	2.9		33.6	±	4.8	27.5	±	5.4		**96.6**	**±**	**6.6**	

Mean and standard deviation of stride velocity, ankle ROM a_ROM, knee ROM k_ROM, hip ROM h_ROM, first peak knee angle in the stance phase k_max1, the minimal knee angle in the late stance phase k_min2 and the second peak knee angle in the swing phase k_max3 for the ipsilateral side in the young (y) and the old (o) age groups. Significant differences are presented in bold. Since stride velocity, step length and cadence were correlated, only stride velocity was tested for significance.

^a^ significant difference from control condition 1 (p < 0.05).

^b^ significant difference from control condition 2 (p < 0.05).

^c^ significant difference from slow control condition (p<0.05).

In the FluMo conditions, the contralateral step length (FluMo 0.61 ± 0.05m, FluMo int. 0.60 ± 0.05m) was slightly larger than the ipsilateral step length (FluMo 0.58 ± 0.04m, FluMo int. 0.57 ± 0.04m).

### Kinematics

**Fig 1:**
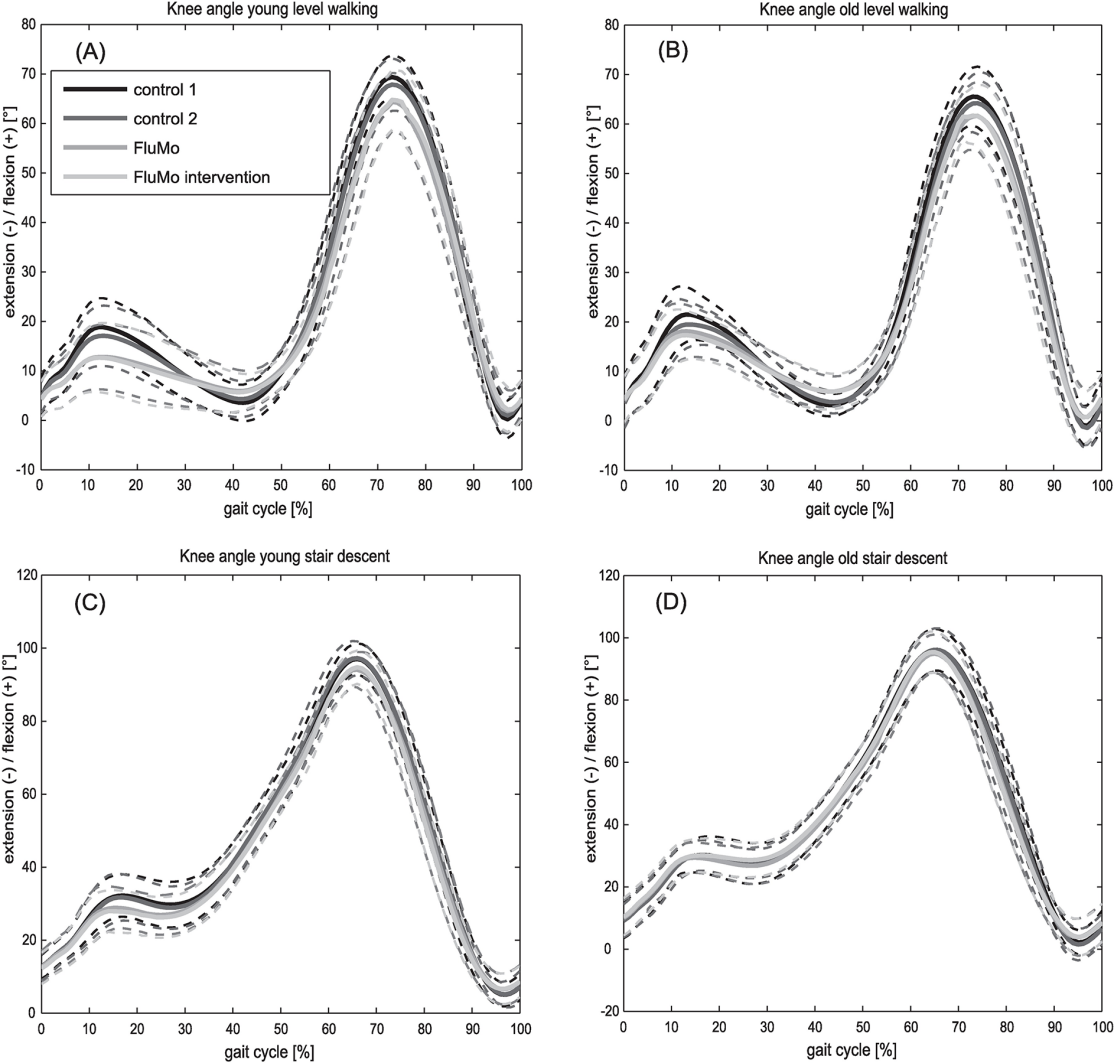
Knee flexion. Mean and standard deviations of knee flexion in level gait for young (A) and elderly (B) subjects, as well as in stair descent for young (C) and elderly (D) subjects, for the conditions control 1, control 2, FluMo and FluMo intervention.

In level gait, all parameters besides the minimal knee angle in the late stance phase *k_min2* were correlated with gait velocity. Regarding the influence of the condition, it was found that the first peak knee angle in the stance phase *k_max1* (within ANCOVA adjusted for gait velocity), did not differ between the conditions (Table 2, Fig 1), except for the old group between the two FluMo and the slow control conditions. But the minimal knee angle in the late stance phase (*k_min2)*, the maximal knee angle in the swing phase *(k_max3)*, as well as *h_ROM*, *k_ROM* and *a_ROM* presented a significant effect of condition (Table 2, Fig 1, S1 Fig, S2 Fig). A post-hoc analysis showed that *a_ROM* and *k_ROM* were significantly lower and *k_min2* significantly higher during the FluMo conditions compared to the two control as well as to the slow control condition (Table 2). For *h_ROM* and *k_max3*, the FluMo condition showed a significantly lower ROM compared to the control as well as the slow control condition, whereas the FluMo intervention condition only differed significantly in comparison to the control 1 and 2 conditions, but not to the slow control condition, although both parameters were adjusted for velocity within the ANCOVA (Table 2).

In stair descent, only *k_ROM* and *h_ROM* were velocity dependent. *k_ROM* and *h_ROM* (adjusted for velocity within ANCOVA), *a_ROM*, the maximal knee flexion angle in the stance phase *(k_max1)* and the minimum knee flexion angle in the late stance phase (*k_min2)* did not differ between any of the conditions (Table 2, S1 Fig, S2 Fig). Furthermore, *k_max3* was affected by the factor condition, revealing a significantly lower maximal flexion in the FluMo than in the control conditions during stair descent (Table 1).

### Ground reaction forces

**Fig 2:**
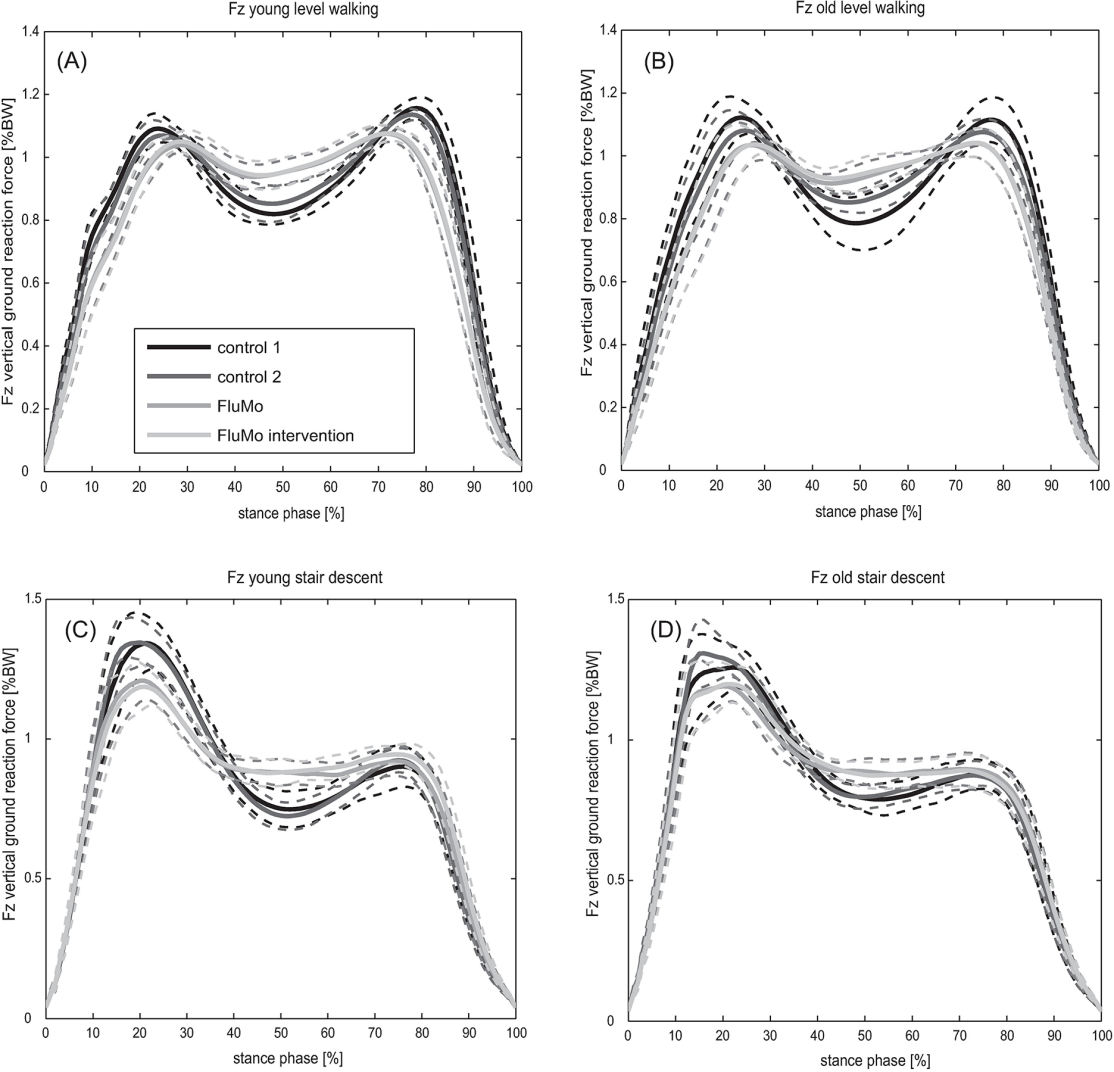
Vertical ground reaction forces. Mean and standard deviation of vertical ground reaction forces (F_z_) in level gait for young (A) and elderly (B) subjects as well as in stair descent for young (C) and elderly (D) subjects for the conditions control 1, control 2, FluMo and FluMo intervention.

In level gait, as well as in stair descent all ground reaction force parameters correlated with gait velocity. Regarding condition comparison, neither for *F*_*z2*_, which was within ANCOVA adjusted for velocity and tested in level gait, as well as stair descent, nor *F*_*ymin*_, which was within ANCOVA adjusted for velocity and tested in stair descent only, exhibited any significant differences between the conditions (Table 3, Fig 2, S3 Fig).

**Table 3:** Ground reaction force parameters.

task	condition	group	F_z2_ [%BW]			F_z3_ [%BW]			F_z4_ [%BW]			b_n_ [%BW]			e_n_ [%BW]			F_ymin_ [%BW]			F_ymax_ [%BW]		
level gait	Control 1	y	1.10	±	0.04	0.81	±	0.03	1.17	±	0.03	8.90	±	0.68	-9.43	±	1.08	-0.18	±	0.02	0.20	±	0.03
		o	1.14	±	0.06	0.77	±	0.08	1.12	±	0.07	8.49	±	1.69	-8.90	±	1.47	-0.19	±	0.04	0.21	±	0.03
	Control 2	y	1.09	±	0.04	0.84	±	0.06	1.14	±	0.01	8.01	±	1.35	-8.43	±	0.68	-0.16	±	0.02	0.18	±	0.02
		o	1.10	±	0.05	0.84	±	0.02	1.08	±	0.04	7.35	±	1.69	-7.58	±	0.91	-0.17	±	0.03	0.18	±	0.02
	FluMo	y	1.06	±	0.02	0.92	±	0.04	1.08	±	0.02	5.73	±	0.80	-5.81	±	1.00	-0.14	±	0.02	0.12	±	0.02
		o	1.06	±	0.06	0.90	±	0.03	1.05	±	0.04	5.62	±	1.34	-6.00	±	1.15	-0.15	±	0.04	0.13	±	0.03
	FluMo int.	y	1.07	±	0.02	0.93	±	0.04	1.09	±	0.02	5.63	±	0.89	-5.88	±	1.09	-0.14	±	0.02	0.12	±	0.03
		o	1.07	±	0.06	0.91	±	0.04	1.05	±	0.04	5.42	±	1.31	-5.87	±	1.07	-0.15	±	0.04	0.13	±	0.03
	Slow control	y	1.07	±	0.02	0.91	±	0.05	1.09	±	0.03	5.45	±	1.22	-6.30	±	1.18	-0.13	±	0.02	0.14	±	0.03
		o	1.07	±	0.06	0.92	±	0.03	1.04	±	0.04	4.81	±	1.55	-5.63	±	1.18	-0.13	±	0.03	0.14	±	0.03
stair descent	Control 1	y	1.43	±	0.08							7.51	±	1.98				-0.14	±	0.02	0.15	±	0.03
		o	1.42	±	0.14							8.95	±	2.68				-0.15	±	0.02	0.15	±	0.02
	Control 2	y	1.44	±	0.17							8.83	±	1.67				-0.14	±	0.02	0.15	±	0.03
		o	1.42	±	0.16							9.23	±	1.65				-0.15	±	0.03	0.15	±	0.02
	FluMo	y	1.28	±	0.08							8.04	±	1.84				-0.14	±	0.03	0.13	±	0.02
		o	1.30	±	0.13							8.27	±	1.68				-0.15	±	0.02	0.14	±	0.02
	FluMo int.	y	1.25	±	0.12							7.65	±	1.27				-0.14	±	0.03	0.13	±	0.03
		o	1.29	±	0.12							7.86	±	1.95				-0.15	±	0.02	0.13	±	0.02

Mean and standard deviation of the first vertical peak ground reaction force *F*_*z2*_, the minimal vertical ground reaction force between the peaks *F*_*z3*_, the second vertical peak ground reaction force *F*_*z4*_, the loading rate *b*_*n*_, the unloading rate *e*_*n*_, the maximal anterior ground reaction force *F*_*ymin*_ and the maximal posterior ground reaction force *F*_*ymax*_ for the ipsilateral side in the young (y) and the old (o) age groups. In level gait, only *F*_*z2*_ was statistically analysed because all other parameters correlated, whereas in stair descent, *F*_*z2*_ and *F*_*ymin*_ were statistically tested.

### Absolute symmetry index and coefficient of multiple correlation

The ASI in step length cadence and stride velocity were smaller than 10% except for the step length ASI’s of the young group in the FluMo conditions (11.0 ± 4.6% and 11.6 ± 4.9%) and the stride velocity of the young group in the FluMo condition (10.1 ± 6.1%) (Table 4). The ground reaction force parameters showed for all conditions averaged ASI below 4% (Table 4).

**Table 4:** Absolute symmetry indexes (ASI).

condition	group	ASI F_z2_ [%]			ASI F_z3_ [%]			ASI F_z4_ [%]			ASI stride velocity [%]			ASI step length [%]			ASI cadence [%]		
Control 1	y	2.0	±	1.6	2.1	±	1.4	1.6	±	1.4	3.4	±	1.8	2.6	±	2.7	1.8	±	1.2
	o	3.5	±	2.1	1.8	±	1.7	3.0	±	2.6	3.0	±	2.0	8.9	±	12.0	4.3	±	4.9
Control 2	y	1.5	±	1.0	1.1	±	0.9	2.1	±	1.9	4.9	±	2.8	3.5	±	3.2	2.4	±	2.1
	o	2.7	±	1.7	1.6	±	0.8	2.0	±	2.1	3.1	±	2.1	3.1	±	2.6	2.3	±	1.3
FluMo	y	1.3	±	0.9	1.3	±	1.1	2.3	±	1.5	10.1	±	6.1	11.0	±	4.6	5.6	±	4.8
	o	3.1	±	2.0	2.5	±	1.6	2.0	±	1.4	5.7	±	3.1	7.5	±	4.0	3.8	±	2.4
FluMo int.	y	1.1	±	1.2	1.1	±	1.0	2.2	±	1.7	9.7	±	4.7	11.6	±	4.9	4.0	±	2.7
	o	3.2	±	2.6	1.4	±	1.3	2.1	±	3.1	6.1	±	4.7	9.4	±	5.8	3.5	±	2.6
Slow control	y	1.6	±	0.9	1.2	±	1.0	1.8	±	1.1	4.6	±	3.8	9.1	±	15.7	7.3	±	13.7
	o	2.7	±	3.0	1.6	±	1.0	2.0	±	0.8	4.5	±	3.2	4.6	±	4.0	3.6	±	2.7

Mean and standard deviation of ASI for level gait in the young (y) and the elderly (o) age groups.

For all tasks and conditions the CMC were always higher than 98%.

## Discussion

The use of advanced technologies such as the moving fluoroscope for assessing the kinematics of skeletal structures under dynamic and loaded conditions presents an important step in understanding biomechanical interactions of the musculoskeletal system. Before such approaches can be accepted for wider clinical and research usage, however, it is critical to understand the potential role that the measurement technique itself plays on a subject’s kinetic and kinematic patterns. In this study, level gait and stair descent were assessed both with and without accompaniment of the moving fluoroscope to assess whether the moving fluoroscope influences human gait. Moreover, an acoustic intervention was used to investigate whether exclusion of the noise of the motors could reduce any effects of the moving fluoroscope.

The freely chosen level gait stride velocities, the ipsilateral step lengths and the ipsilateral cadences of the control conditions were comparable to standard gait time distance parameters of previous studies [28, 29]. In contrast, the gait velocity, step lengths and cadence of the conditions with the moving fluoroscope were lower than in the control conditions, indicating that the moving fluoroscope altered time distance parameters towards those of the slow walking condition. This is in contrast to the findings of Yamokoski and Banks (14), who found that on average people moved faster through the dynamic radiographic system while the robots were actively following their motion. This might be explained by the fact that in the latter study, subjects walked through the workspace of the robots, while the subjects in the present study walked within the moving fluoroscope. The slower gait velocity for the moving fluoroscope conditions can also partly be explained by the fact that some subjects had to be instructed to reduce the gait velocity to allow good tracking of the moving fluoroscope, due to the limited acceleration of the robot (ca. 8m/s^2^). However, the stride velocities in the FluMo conditions were still in the range of the gait velocities measured in subjects with TKA [30], but slightly lower than the fastest TKA subjects reported within a meta-analysis of Abbasi-Faghi et al. [31]. Interestingly, the second control trials’ velocities were significantly higher than in the FluMo conditions, but significantly lower than in the first control condition suggesting that the subjects were influenced by the previously slower trials of the FluMo conditions. In stair descent, the step length (~ 0.32m) was equal for all conditions due to the defined stair step length. Although the time distance parameters during level gait in general reflected a symmetric gait pattern (ASI < 10%), the contralateral step length was slightly larger (>10% difference [26, 27]) than the ipsilateral step length in the FluMo conditions, suggesting that the step length of the ipsilateral leg might be influenced by the moving fluoroscope. This phenomenon might be partially explained by the somatic perception of the wire sensor that was attached to the ipsilateral leg and the divergent acceleration pattern of the moving fluoroscope during an ipsi- and a contralateral step.

**Fig 3:**
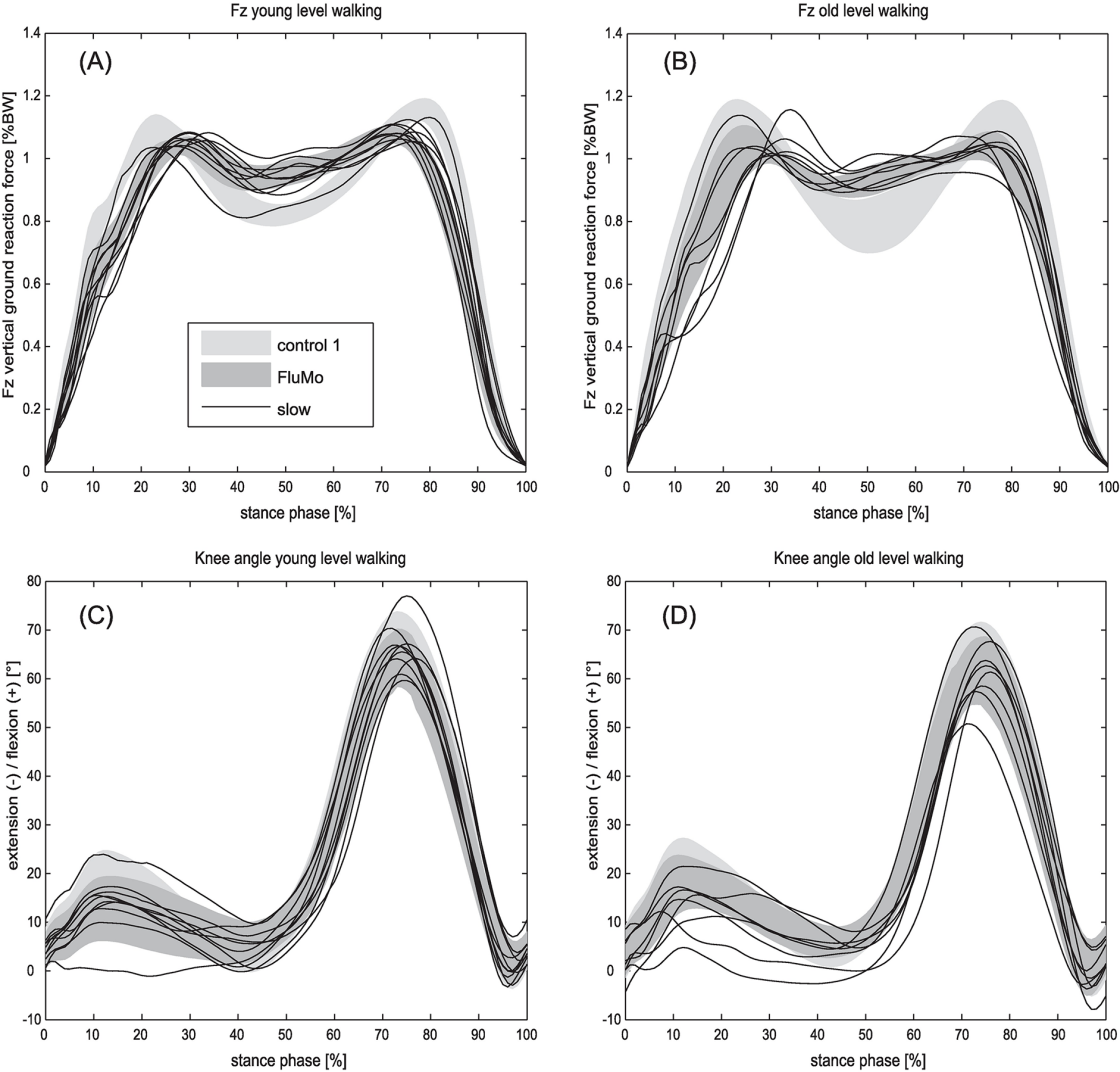
Knee flexion and vertical ground reaction force for the slow in comparison to the FluMo and the control 1 conditions. The grey areas represent the range between the mean plus/minus standard deviation of the vertical ground reaction forces (A, B) and knee flexion (C, D) for young (A, C) and elderly (B, D) subjects for the conditions control 1 and FluMo in level gait. The black lines represent the mean of the two slow gait trials for each subject.

All kinematic parameters of the ankle, knee and hip joints during the control conditions in level gait were similar to past research [23, 32, 33], whereas the FluMo conditions differed in some kinematic parameters from the control conditions. The moving fluoroscope influenced the maximal knee angle in the stance phase only for the old group in comparison to the slow control condition, but not the two control conditions. The maximal knee angle in the swing phase, the minimal knee angle in the late stance phase, as well as the ankle, knee and hip ROMs were significantly different when walking with the moving fluoroscope in comparison to walking without. Although within ANCOVA adjusted for walking velocity, the hip ROM as well as the maximal knee angle in the swing phase of the FluMo intervention condition only differed from the control condition with preferred gait speed but not from the slow gait condition. Therefore, and especially also based on the significant difference between the slow control and the control 1 conditions for the hip and knee ROM, it must be assumed that velocity adjustment by including velocity as a covariate within the statistical analysis did not fully take effect. Comparing kinematic motion characteristics, it can be summarised that walking with the moving fluoroscope is very similar to walking at a slow gait speed (Fig 3).

Since it is well known that gait velocity has an influence on the kinematic parameters [34], velocity dependent parameters were within ANCOVA adjusted for walking speed to allow for condition comparisons. However, since velocity adjustment within the linear mixed factors ANCOVA is based on the assumption of a linear regression and not all parameters exhibit a linear velocity dependency, a comparison of the kinematic parameters is somewhat limited in its validity. The efficacy of linear velocity adjustment especially seemed to fail for parameters that showed a significant difference between the FluMo conditions and the control 1 and 2 conditions, but not the slow control conditions, or between the slow control and the control 1 condition, although they were adjusted for velocity within the ANCOVA (e.g. *k_max3*, *h_ROM*). However, since a predefined gait velocity may have led to large adaptations in gait characteristics, the measurements of the control conditions with the self-selected gait speed still seem to be the preferential methodology. This study limitation needs to be kept in mind since it complicates the interpretation of the kinematic and kinetic parameters.

In stair descent, the ankle ROM was not influenced by the moving fluoroscope, but was slightly larger for all conditions than the values reported by Riener et al. [35]. The knee ROM as well as the first peak knee angle in the stance phase were comparable to the previous literature [35]. However, the moving fluoroscope did show a significant effect on the maximal flexion angle in the swing phase. While the maximal flexion angle in the swing phase was significantly different to both control conditions, it should be noted that e.g. the difference for the maximal knee flexion angle between the FluMo and the control 2 condition was for 9 subjects below 1°, for 3 subjects between 1° and 2°, for 5 subjects between 2° and 3° and for two subjects between 3 and 4°, while the inter-trial standard deviation of the individual subjects for the maximal flexion angle was up to 3.5° for the FluMo and 3.6° for the control 2 condition. Thus, although a significant influence in the latter parameter was found, the difference was in the range of the inter-trial variation of the individual subjects as well as the actual error that can be expected within skin marker measurements due to soft tissue artefacts [36, 37].

The vertical ground reaction forces in level gait exhibited a typical “m” shape with a first peak, an unloading phase and a second peak (Fig 2). All ground reaction force parameters were comparable to existing literature [25], except for the minimal vertical ground reaction force between the peaks, which was smaller in all conditions than the values presented by Stacoff and co-workers [25]. However, dependency of kinetic parameters on gait velocity, as observed in the larger first and second peaks of the vertical ground reaction forces and reduced unloading in between, when comparing the slow level gait and the FluMo conditions to the control conditions, is in agreement with previous studies [38]. The ground reaction force characteristics, as well as the gait velocity of the slow condition, were similar to the FluMo condition (Fig 3). Since the ground reaction force parameters, when adjusted for velocity within the ANCOVA, did not show any difference between walking with, compared to walking without the moving fluoroscope, it can be concluded that ground reaction force characteristics when walking with the moving fluoroscope are similar to natural slow gait.

In this study, inter-limb symmetry was evaluated based on the vertical ground reaction forces. According to a previous study of Stacoff et al. [25], asymmetric gait patterns can be detected when ASI of the vertical ground reaction force parameters exceed 5%. Since the ASI for the ground reaction force parameters of the present study were below 4%, even when walking with the moving fluoroscope, it can be concluded that the subjects did not show unnatural gait patterns like limping when walking with the moving fluoroscope (Table 4).

Similarly, in stair descent, the first peak of the vertical ground reaction force, as well as the maximal anterior ground reaction force, both adjusted for gait velocity within the ANCOVA, did not show a significant impact of the condition (Table 3). Therefore, it can be concluded that the moving fluoroscope causes no change in the investigated kinetic parameters when compared to walking at a similar gait speed. Since no differences were found between the FluMo and the FluMo intervention condition in both tasks, it can be assumed that the sound of the moving fluoroscope did not influence the subjects during either level walking or stair descent. Since some kinematic parameters were indeed influenced when walking with the moving fluoroscope, other influences such as the appearance of the device, the wire sensor, and visual information such as the movement of the c-arm may have an influence on the subject’s gait. Further improvement of the moving fluoroscope should therefore aim to replace the wire sensor with a tracking methodology that does not require direct contact with the subject.

## Conclusions

To conclude, walking with the moving fluoroscope leads to a decrease in gait velocity compared to the control conditions, however gait with the moving fluoroscope did not differ from natural gait for all ground reaction force parameters when adjusted for differences in gait velocity. However, since some kinematic parameters were influenced, it cannot be completely excluded that at least some subjects were influenced by the moving fluoroscope, but statistical comparison in this respect was limited due to velocity differences between the conditions. Overall, gait characteristics in the natural gait condition were comparable to the conditions with the moving fluoroscope. However, walking with the moving fluoroscope is restricted to a limited gait speed, especially for young subjects. For subjects with TKA showing slower gait velocities [30], the speed capability of the moving fluoroscope seems to be reasonable. To assess gait at standard gait velocities of healthy subjects, the moving fluoroscope would need to be optimized in order to allow higher accelerations to occur at higher gait velocities.

To conclude, the moving fluoroscope is suitable for comparisons between groups measured with the moving fluoroscope, such as e.g. comparisons between implant designs and healthy knees. However, care should be taken when comparing data to subjects walking at self-selected speed without the moving fluoroscope. Here, correction due to gait velocity differences should be treated with care, especially in relation to joint angles.

## Supporting information

S1 Fig Ankle sagittal plane movement. Mean and standard deviation of ankle sagittal plane movement in level gait (A, B) and stair descent (C, D) for the young (A, C) and elderly (B, D) age groups.
(TIF)

S2 Fig Hip sagittal plane movement. Mean and standard deviation of hip flexion/extension in level gait (A, B) and stair descent (C, D) for the young (A, C) and elderly (B, D) age groups.
(TIF)

S3 Fig Anteroposterior ground reaction forces. Mean and standard deviation of anterior (negative) and posterior (positive) ground reaction forces (F_y_) in level gait (A. B) and stair descent (C, D) for the young (A, C) and elderly (B, D) age groups.
(TIF)
