# Supplementary figures and images for: Influence of the moving fluoroscope on gait patterns
Source: PLoS One. 2018 Jul 13;13(7):e0200608. doi: 10.1371/journal.pone.0200608 (PMC6044540; doi:10.1371/journal.pone.0200608)

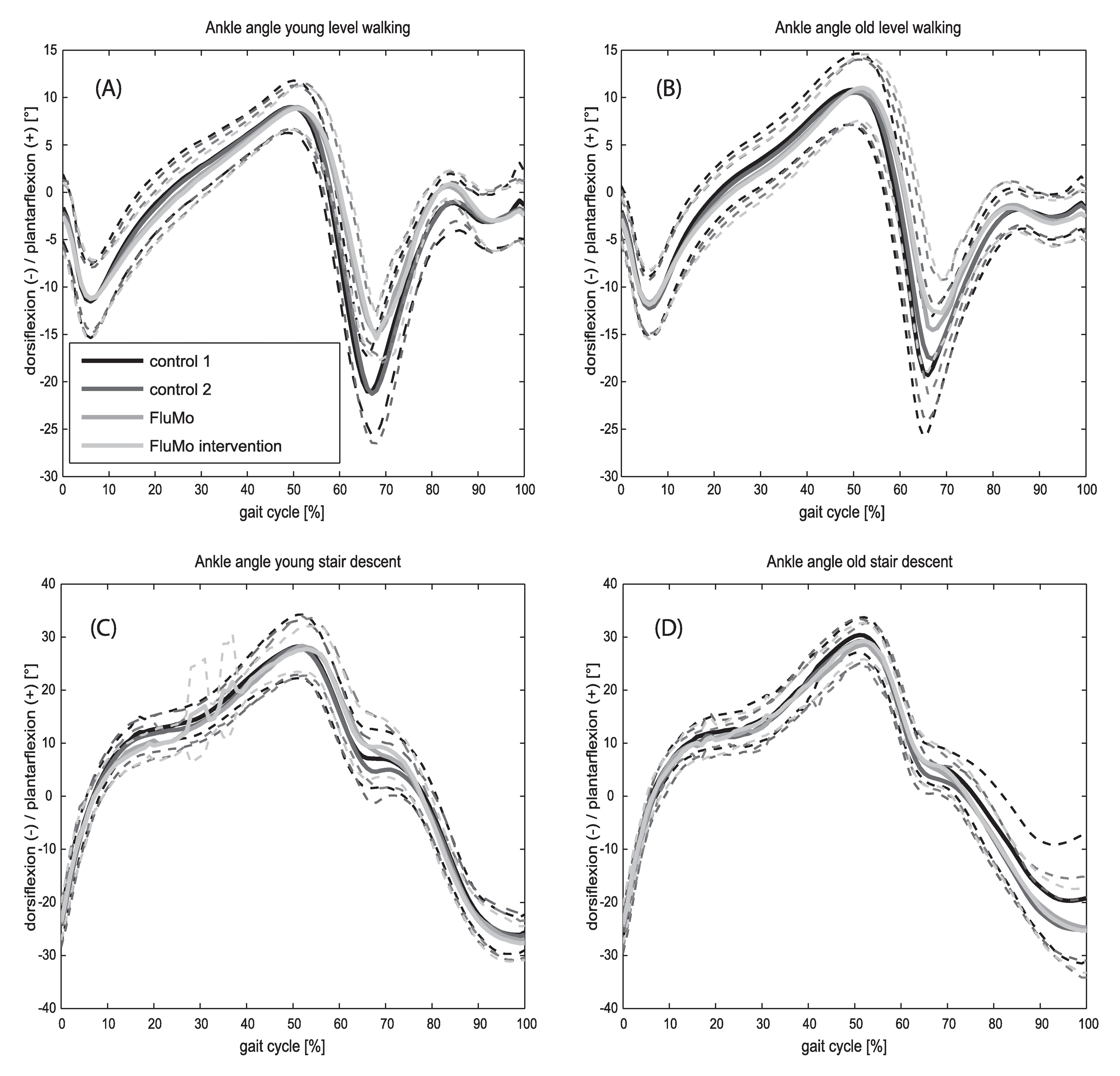

Supplement: S1 Fig — Mean and standard deviation of ankle sagittal plane movement in level gait (A, B) and stair descent (C, D) for the young (A, C) and elderly (B, D) age groups. (TIF) [file pone.0200608.s001.tif]

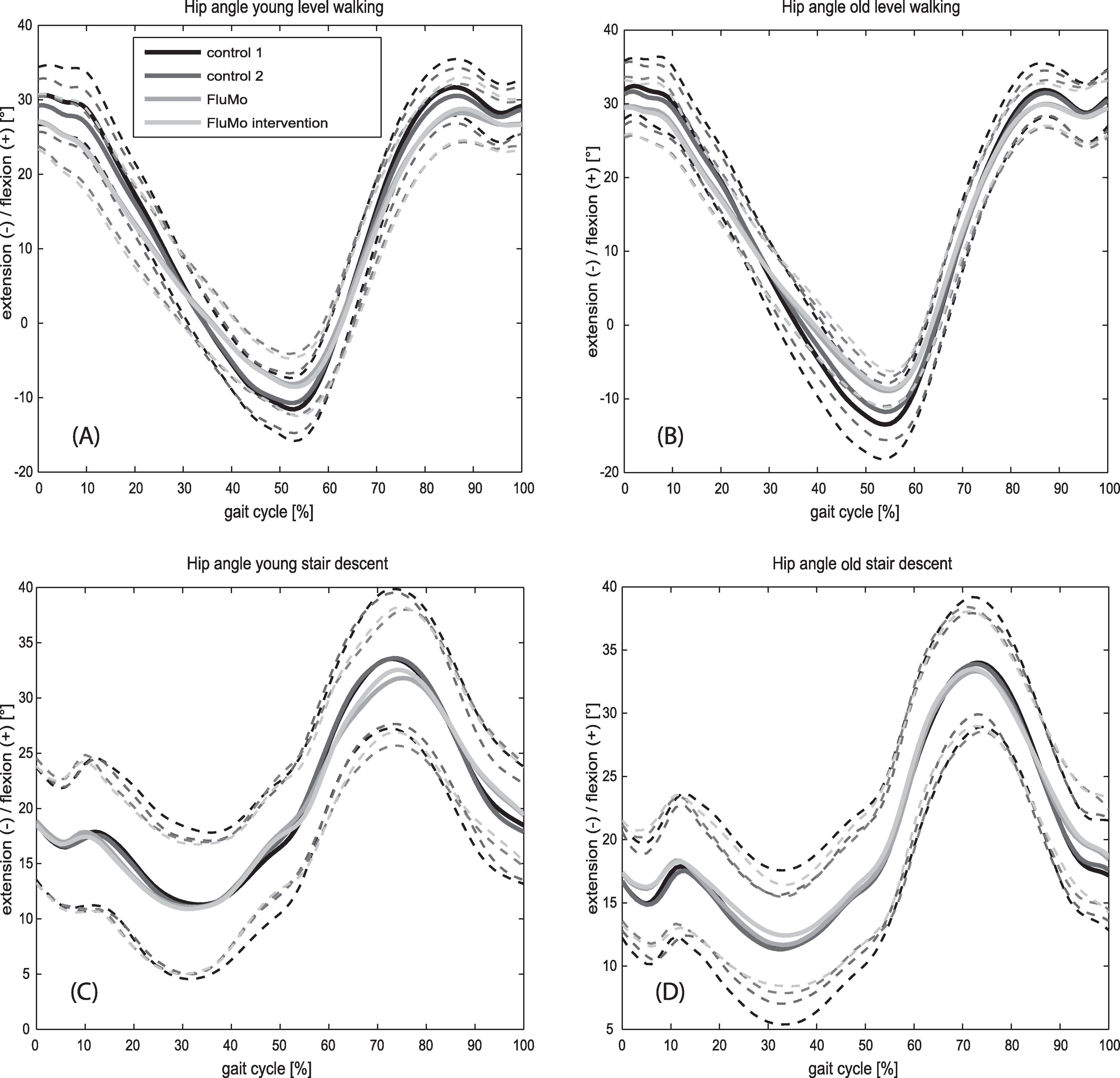

Supplement: S2 Fig — Mean and standard deviation of hip flexion/extension in level gait (A, B) and stair descent (C, D) for the young (A, C) and elderly (B, D) age groups. (TIF) [file pone.0200608.s002.tif]

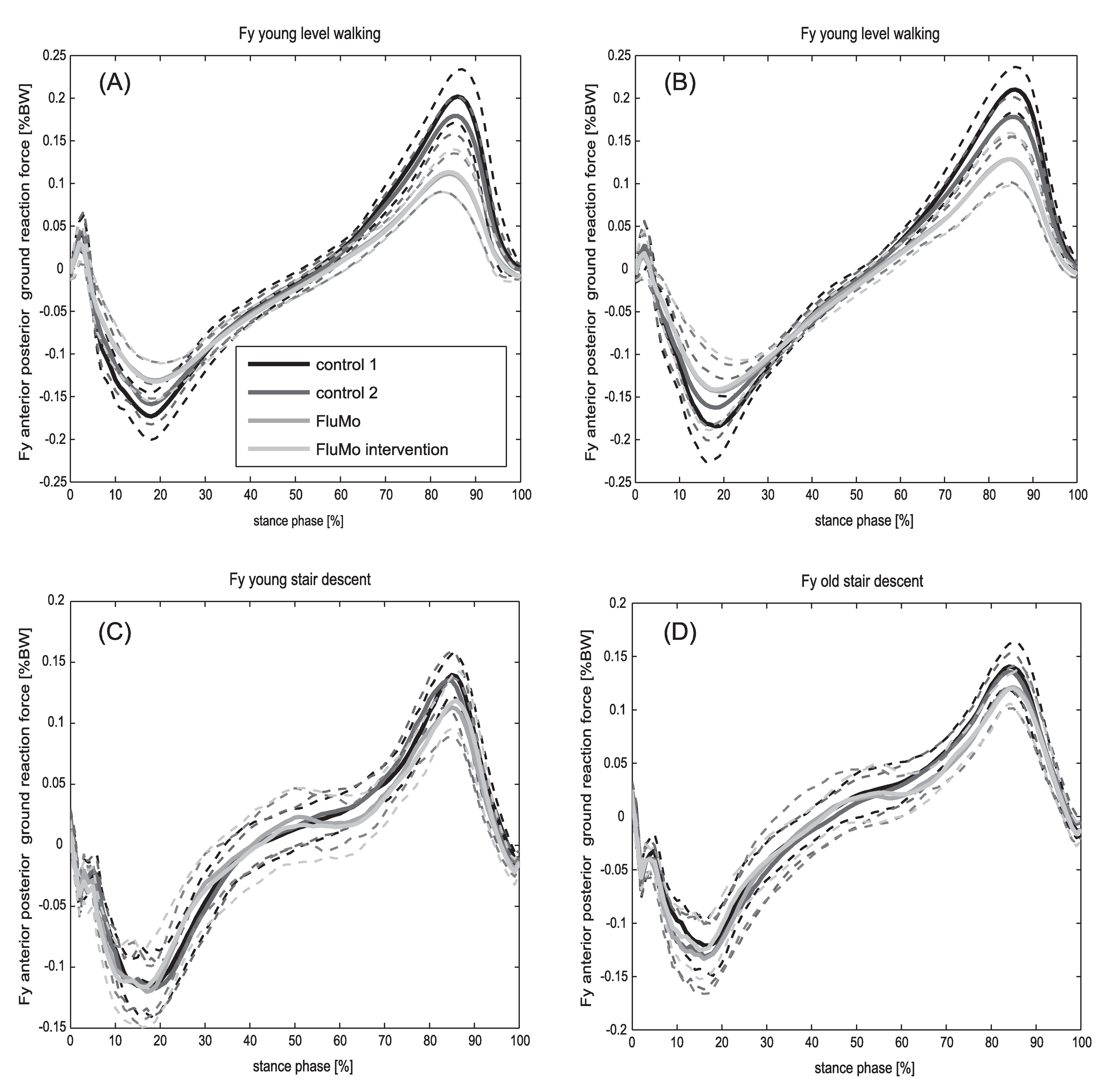

Supplement: S3 Fig — Mean and standard deviation of anterior (negative) and posterior (positive) ground reaction forces (Fy) in level gait (A. B) and stair descent (C, D) for the young (A, C) and elderly (B, D) age groups. (TIF) [file pone.0200608.s003.tif]
